# Cerebral O_2_ and CO_2_ transport in isovolumic haemodilution: Compensation of cerebral delivery of O_2_ and maintenance of cerebrovascular reactivity to CO_2_

**DOI:** 10.1177/0271678X221119442

**Published:** 2022-09-21

**Authors:** Jay MJR Carr, Philip N Ainslie, David B MacLeod, Joshua C Tremblay, Daniela Nowak-Flück, Connor A Howe, Mike Stembridge, Alexander Patrician, Geoff B Coombs, Benjamin S Stacey, Damian M Bailey, Daniel J Green, Ryan L Hoiland

**Affiliations:** 1 Centre for Heart, Lung and Vascular Health, University of British Columbia – Okanagan Campus, School of Health and Exercise Sciences, Kelowna, B.C., Canada, V1V 1V7; 2 Human Pharmacology & Physiology Lab, Department of Anesthesiology, Duke University Medical Center, Durham, NC, USA; 3 Cardiff School of Sport and Health Sciences, Cardiff Metropolitan University, Cardiff, UK; 4 School of Kinesiology, Faculty of Health Sciences, University of Western Ontario, London, Ontario, Canada; 5 Neurovascular Research Laboratory, Faculty of Life Sciences and Education, University of South Wales, Pontypridd, UK; 6 School of Human Sciences (Exercise and Sport Sciences), The University of Western Australia, Nedlands, Western Australia; 7 Department of Anesthesiology, Pharmacology and Therapeutics, Faculty of Medicine, Vancouver General Hospital, University of British Columbia, Vancouver, BC, Canada; 8 Department of Cellular and Physiological Sciences, Faculty of Medicine, University of British Columbia, Vancouver, BC, Canada; 9 International Collaboration on Repair Discoveries (ICORD), University of British Columbia, Vancouver, British Columbia, Canada

**Keywords:** Hypoxaemia, hypercapnia, haemoglobin, anaemia, cerebral blood flow, nitric oxide

## Abstract

This study investigated the influence of acute reductions in arterial O_2_ content (CaO_2_) via isovolumic haemodilution on global cerebral blood flow (gCBF) and cerebrovascular CO_2_ reactivity (CVR) in 11 healthy males (age; 28 ± 7 years: body mass index; 23 ± 2 kg/m^2^). Radial artery and internal jugular vein catheters provided measurement of blood pressure and gases, quantification of cerebral metabolism, cerebral CO_2_ washout, and trans-cerebral nitrite exchange (ozone based chemiluminescence). Prior to and following haemodilution, the partial pressure of arterial CO_2_ (PaCO_2_) was elevated with dynamic end-tidal forcing while gCBF was measured with duplex ultrasound. CVR was determined as the slope of the gCBF response and PaCO_2_. Replacement of ∼20% of blood volume with an equal volume of 5% human serum albumin (Alburex® 5%) reduced haemoglobin (13.8 ± 0.8 vs. 11.3 ± 0.6 g/dL; P < 0.001) and CaO_2_ (18.9 ± 1.0 vs 15.0 ± 0.8 mL/dL P < 0.001), elevated gCBF (+18 ± 11%; P = 0.002), preserved cerebral oxygen delivery (P = 0.49), and elevated CO_2_ washout (+11%; P = 0.01). The net cerebral uptake of nitrite (11.6 ± 14.0 nmol/min; P = 0.027) at baseline was abolished following haemodilution (−3.6 ± 17.9 nmol/min; P = 0.54), perhaps underpinning the conservation of CVR (61.7 ± 19.0 vs. 69.0 ± 19.2 mL/min/mmHg; P = 0.23). These findings demonstrate that the cerebrovascular responses to acute anaemia in healthy humans are sufficient to support the maintenance of CVR.

## Introduction

Carbon dioxide (CO_2_) and, to a lesser extent, oxygen (O_2_) are amongst the most potent regulators of cerebral blood flow (CBF) in humans.^1,2^ Increases and decreases in the partial pressure of arterial CO_2_ (PaCO_2_) elicit concomitant changes in CBF,^2,3^ in order to maintain cerebral tissue “washout” of CO_2_ and regulate cerebral acid-base balance.^4–9^ Reductions in the partial pressure of arterial O_2_ (PaO_2_) elevate CBF^2,3,10^ in order to sustain cerebral delivery of O_2_ (CDO_2_);^11^ while reductions of arterial O_2_ content (CaO_2_: anaemic hypoxia) independent of PaO_2_, also augment CBF^12–16^ or O_2_ extraction^17,18^ such that metabolism is maintained. In short, these homeostatic responses to changes in CO_2_ and O_2_ act to regulate conditions appropriate for stable cerebral metabolism.

Relative to reductions of CaO_2_ independent of PaO_2_, anaemia is a hallmark of multiple disease states with well-documented cerebrovascular dysregulation, including sickle cell disease^19,20^ and chronic renal failure.^21–23^ For example, sickle cell disease increases the risk of stroke, which may be related to a reduction in cerebrovascular vasodilatory capacity^19,20,24^ secondary to elevations in resting CBF.^19,20,24^ Similarly, CVR is reduced in chronic kidney disease patients with anaemia concomitant to elevations in resting CBF.^21^ While pre-clinical studies have demonstrated that brain parenchymal O_2_ tension, and CBF responses to hypercapnia are preserved during subacute anaemia in rats through elevated basal CBF,^25^ CDO_2_ is impaired in human chronic kidney disease patients despite similarly increased CBF.^22^ Whether the differences in CBF regulation observed with anaemia are related to anaemia *per se* or a consequence of the disease state underlying said anaemia remains to be determined.

Work in healthy humans assessing the effects of isovolumic haemodilution on functional responses in the peripheral circulation has demonstrated increased endothelium-mediated vasodilation (i.e. brachial artery reactive hyperemia flow-mediated dilation;^26,27^) and maintained O_2_ delivery to working skeletal muscles.^28,29^ Furthermore, in healthy newborn baboons, CVR varies inversely with hematocrit (Hct) after haemodilution and haemoconcentration.^30^ Whether the slope of the CBF response to changes in CO_2_ [cerebrovascular reactivity (CVR)] would be steepened during acute anaemia in healthy humans given the absence of pathology associated vascular dysfunction remains unknown. Based on the aforementioned evidence, anaemia – in the absence of pathology related impairments in vasodilatory signalling^31^ – may lead to a steepening of CO_2_ CVR due to a reduction in haemoglobin (Hb)-mediated nitric oxide (NO) scavenging.^26,27,32^ Given the complex roles of NO^33–36^ and cerebral acid-base balance^4–9^ in cerebrovascular regulation we quantified blood-borne markers of NO uptake/release, cerebral metabolism, and cerebral CO_2_ washout to provide insight into the influence of anaemia on CVR. We hypothesized that acute experimental haemodilution in healthy humans would increase CBF and CVR, while CDO_2_, CO_2_ washout, and cerebral metabolism would be preserved.

## Methods

### Ethical approval

This study was conducted in the Centre for Heart, Lung and Vascular Health at the University of British Columbia Okanagan and adhered to the standards outlined in the Declaration of Helsinki (except registry in a database) and the Canadian Tri-council Policy Statement for Integrity in Research. Institutional approval was received from the University of British Columbia Clinical Research Ethics Board (CREB ID: H16-01028). All participants provided written informed consent prior to participation in this study.

### Participants

Eleven healthy males were recruited to participate in this study (age; 28 ± 7 years: body mass index; 23 ± 2 kg/m^2^). Participants attended the laboratory at 0600 hr or 1300 hr having abstained from alcohol, caffeine, and exercise for 24 hours, fasted for at least 4 hours, but drank water *ad libitum*. All participants were free of overt cardiovascular, cerebrovascular, respiratory, neurological, and metabolic disease, and all were non-smokers with no history of smoking, and not taking prescription medicine related to any aforementioned conditions.

### Experimental protocol

Participants rested in the supine position upon arrival and were instrumented with radial artery, antecubital vein, and internal jugular vein catheters. Using sterile technique, a 20 G arterial catheter (Arrow, Markham, ON) was advanced into the left radial artery under local anesthesia (Lidocaine, 1.0%). This technique was assisted via ultrasound guidance. A 13 G central venous catheter (Cook Medical, Bloomington, IN) was advanced into the right internal jugular vein under sterile conditions and local anesthesia with ultrasound guidance. The catheter was then advanced up to 15 cm cephalad.^37^ The same technique, performed by our group has been previously demonstrated to lead to catheter tip placement in the jugular bulb, superior to the facial vein.^11^ Further, correct placement was additionally determined by participants noting a sensation in their ear upon full insertion of the catheter.^37^ Fulfilment of these techniques leads to ≤3% contamination by extra-cerebral blood.^37^ Finally, an 18 G venous catheter (Insyte™ Autoguard™, Becton Dickinson, USA) was inserted into the median antecubital vein. Following catheterization, the remainder of the monitoring equipment was set-up while the participant then rested quietly for 20 minutes (see below).

Using dynamic end-tidal forcing (DEF), subjects completed a stepwise steady-state hypercapnic test to assess CVR both pre- and post- an isovolumic haemodilution protocol. Following five minutes of steady room-air breathing to assess baseline P_ET_CO_2_ and P_ET_O_2_ values, subjects breathed for two minutes with DEF control at baseline end-tidal values. Then P_ET_CO_2_ was elevated in three sequential step-wise increments to +3, +6, and +9 mmHg P_ET_CO_2_ above baseline. Each stage of elevated P_ET_CO_2_ lasted 5-minutes following the attainment of steady end-tidal gases (three breaths within ±1mmHg of target P_ET_CO_2_) and all cerebrovascular, haemodynamic, cardiorespiratory variables, and arterial blood samples were collected in within the last 1–2 minutes of each stage following attainment of physiologic steady state.^38^ Following completion of the test, participants returned to room air breathing.

After pre-haemodilution CVR testing to reduce hematocrit and thereby reduce CaO_2_, whole blood was transferred into an Anticoagulant Citrate Phosphate Dextrose Solution BLOOD-PACK™ (4R0012MC, Fenwal, USA) through a non-pyrogenic plasma transfer kit (4C2240, Fenwal, USA) to collect up to 450 mL of whole blood per BLOOD-PACK for short term storage at room temperature on an orbital agitator. This blood was replaced with an equal volume of 5% human serum albumin (Alburex® 5%) in two increments that amounted to a total removal and replacement of ∼20% of blood volume. The magnitude of haemodilution was verified by measuring the resulting change in hematocrit. Following haemodilution, CVR was re-conducted (see supplemental Figure 1 for protocol schematic).

## Experimental measures

### Cardiorespiratory measures

All cardiorespiratory variables were sampled continuously at 1 KHz via an analogue-to-digital converter (Powerlab, 16/30; ADInstruments, Colorado Springs, CO). Heart rate (HR; ADI bioamp ML132), radial artery blood pressures, and jugular venous blood pressures (Truwave Transducer, Edwards Life Sciences) were measured as previously described.^11^ The partial pressures of end-tidal CO_2_ (P_ET_CO_2_) and O_2_ (P_ET_O_2_) were sampled at the mouth and recorded by a calibrated gas analyzer (model ML206, ADInstruments), while respiratory flow was measured by a pneumotachograph (MLT1000L; ADInstruments) paired with a spirometer (FE141; ADInstruments) and connected in series to a bacteriological filter. All data were interfaced with LabChart (Version 7).

### Cerebral vascular measurements

Diameter and blood velocity of the right internal carotid artery (ICA) and left vertebral artery (VA) were measured using a 10 MHz multi-frequency linear array duplex ultrasound (Terason uSmart 3300, Teratech, Burlington, MA). Specifically, B-mode imaging was used to measure arterial diameter, while pulse-wave mode was used to concurrently measure blood velocity. Diameter and velocity of the ICA were measured at least 1.5 cm distal to the common carotid bifurcation to eliminate recordings of turbulent and retrograde flow and non-uniform shear. The VA blood velocity and diameter were measured between C4 and C5 or C5 and C6. Care was taken to ensure that the insonation angle (60°) was unchanged throughout each test. Further, upon acquisition of the first ultrasound image (i.e., resting baseline) there was no alteration of B-mode settings to avoid any artificial changes in arterial wall brightness/thickness. All recordings were made in accordance with published technical recommendations.^39^ Volumetric blood flow in the ICA and VA were quantified using the following equation:

$$\mathrm{ICA}\;or\;VA\;\mathrm{flow}=\left(\pi \cdot {\left(\frac{\mathrm{diameter}\;(cm)}{2}\right)}^{2}\right)\cdot \frac{\mathrm{peak}\;\mathrm{envelope}\;\mathrm{velocity}\;\left(\frac{cm}{s}\right)}{2}\;\cdot \frac{60\;s}{\mathrm{minute}}$$

Volumetric global cerebral blood flow (gCBF) was subsequently calculated as:

$$\mathrm{gCBF}=\left(\mathrm{ICA}\;\mathrm{flow}+VA\;\mathrm{flow}\right)\cdot 2$$

Blood velocity in the right middle cerebral artery (MCAv) and left posterior cerebral artery (PCAv) were measured using a 2 MHz transcranial Doppler ultrasound (TCD; Spencer Technologies, Seattle, WA). The TCD probe was fixed to a headpiece (model M600 bilateral head frame, Spencer Technologies) and secured into place. The MCA and PCA were insonated through the middle trans-temporal window, using previously described location and standardization techniques.^40^

All recordings were screen captured and stored as video files for offline analysis. This analysis involved concurrent determination of arterial diameter and peak blood velocity at 30 Hz, using customized edge detection and wall tracking software (BloodFlow Analysis, Version 5.1) designed to mitigate observer bias.^41^

#### Blood sampling & analyses

For the measurement of arterial blood gases, ∼1.0 mL of radial arterial blood was drawn into a pre-heparinized syringe (SafePICO, Radiometer, Copenhagen, Denmark) and analyzed immediately at 37°C using a commercial blood gas analyzer (ABL90 FLEX, Radiometer). This analysis included measurement of the partial pressure of arterial oxygen (PaO_2_), PaCO_2_, arterial oxygen saturation (SaO_2_), arterial oxygen content (CaO_2_), [Hb] and Hct. Arterial blood was also analyzed for whole blood viscosity. Arterial blood for viscosity measurement was drawn into a Lithium Heparin Vacutainer® (Becton Dickinson, USA). Blood viscosity was measured in duplicate within 15 min of blood sample acquisition at three shear rates (45, 90, and 225 s^−1^) at 37.0°C with a temperature controlled cone-and-plate viscometer (Model DV2T; Brookfield Amtek, Middleboro, MA, USA).^42^

For NO analyses, arterial and jugular bulb blood samples were drawn into K_2_EDTA Vacutainers® (Becton Dickinson, USA). Whole blood was then immediately centrifuged at 600 g for 10 minutes at a temperature of 4.0°C. Plasma was aliquoted into cryovials, flash frozen in liquid N_2_, and stored at −80°C.

Ozone-based chemiluminescence (Sievers NOA™ 280i, Analytix Ltd, Durham, UK) was employed to measure plasma ${\mathrm{NO}}_{2}^{-}$ via chemical reagent cleavage. A detailed overview of the methods including principles of detection has previously been published.^33,43,44^ Plasma was mixed with 5% acidified sulphanilamide and left to incubate in the dark at 21°C for 15 min to remove ${\mathrm{NO}}_{2}^{-}$ prior to injection into tri-iodide reagent for direct measurement of *S*-nitrosothiols (RSNO). A separate sample was also injected into tri-iodide reagent for the combined measurement of ${\mathrm{NO}}_{2}^{-}$ and RSNO with ${\mathrm{NO}}_{2}^{-}$ calculated by subtracting the concentration of RSNO. Signal output (mV) was plotted against time (s) using Origin 8 software (OriginLab Corps, Massachusetts, USA) and smoothed using a 150-point averaging algorithm. The Peak Analysis package was used to calculate the area under the curve (mV/s) and subsequently converted to a concentration, using standard curves of known concentrations of sodium nitrite. The intra- and inter-assay coefficient of variations for all metabolites were *<*10%.

### Statistical analyses

Hematological, blood gas, and cerebral metabolism parameters at rest pre and post haemodilution were compared using two-tailed paired t-tests. Blood gas parameters for the hypercapnia trials pre and post haemodilution were analyzed with linear mixed effects models. Trial (pre/post haemodilution) and stage (baseline, +3, +6, +9 PaCO_2_) were included as fixed effects while *participants* were included as a random effect. When significant interaction effects were detected, post-hoc tests were conducted with multiple comparisons adjusted for with the Bonferroni correction.

All CBF (VA flow, ICA flow, & gCBF) and shear stress responses to hypercapnia pre- and post-haemodilution were analyzed with linear mixed effects models (Fixed effects: PaCO_2_ and pre/post haemodilution; Random effect: *participants*). When significant interaction effects were detected, post-hoc tests were conducted with multiple comparisons adjusted for with the Bonferroni correction. As the haemodilution protocol would be expected to cause a set-point change in absolute CBF due to the well-established relationships between CaO_2_, [Hb], and CBF (e.g., Brown et al., 1985), we also analyzed CBF responses by assessing the delta changes in CBF from baseline in each trial. This approach was taken to account for the elevation in CBF that occurred secondary to haemodilution, but prior to the hypercapnia. The change in CBF and shear stress from baseline (i.e. delta values) were compared using linear mixed effects model with trial (pre/post haemodilution) and stage (baseline, +3, +6, +9 PaCO_2_) as fixed effects. *Participants* were included as a random effect. When significant interaction effects were detected, post-hoc tests were conducted with multiple comparisons adjusted for with the Bonferroni correction. Further, CVR slopes were calculated using linear regression and compared pre- to post-haemodilution with two tailed paired t-tests.

To support existing literature on the relationships between CBF and: CaO_2_, Hb, Hct, PaCO_2_, pH, and CPP, we conducted repeated measures correlations using the rmcorr package for R^45^ on those variables at three time points (pre-haemodilution baseline, mid-haemodilution check-point, and post haemodilution baseline), to quantify the intra-individual relationships between those variables and CBF.

To determine whether significant a–v differences and net exchange (uptake/release) of ${\mathrm{NO}}_{2}^{-}$ was present before or after haemodilution we conducted one-sided two-tailed t-tests (tested against a zero value) within each condition. We also compared the a–v difference and cerebral net exchange of ${\mathrm{NO}}_{2}^{-}$ prior to and following haemodilution using a two-tailed paired t-test.

Linear mixed model analyses were performed with the Statistical Package for the Social Sciences (SPSS, Version 24), t-tests were conducted using Excel (Microsoft, Version 16.16.9), and repeated measures correlations were conducted using R.^46^ All figures were generated using GraphPad (Prism, La Jolla, CA, Version 8.1.1). Shapiro–Wilk normality testing of the fundamental stimuli (PaCO_2_, PaO_2_, CaO_2_) and measured responses upon which conclusions were based (ICA flow, VA flow, gCBF), confirmed that the data were normally distributed.

Statistical significance was set *a priori* at P < 0.05 and actual P values are reported herein (unless P < 0.001).^47,48^ This invasive study resulted in a relatively small sample size, as such there may be an increased risk of type 2 error, and we conduct multiple comparisons which in itself may predispose to an increased risk of type 1 error; so, in addition to significance testing, individual data are displayed for the sake of transparency and improved ease of data interpretation, of particular utility where sample sizes are low and P-values approach the P = 0.05 cut-off. Data are presented as means and standard deviations.

## Results

### Baseline, pre and post haemodilution

#### Intervention parameters

**Figure 1.**
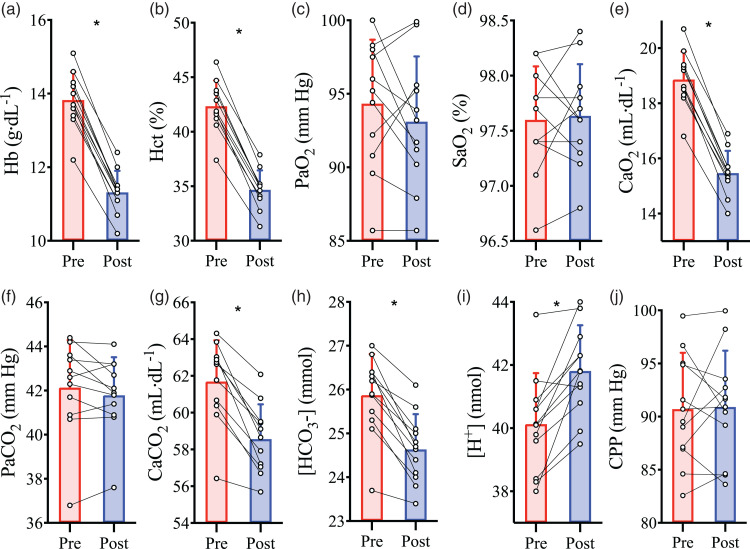
Intervention parameters at baseline prior to and following haemodilution. Pre-haemodilution mean and standard deviation data presented in red, post-haemodilution mean and standard deviation data presented in blue, with individual data overlaid for both. (a) haemoglobin concentration ([Hb]), (b) hematocrit (Hct), (c) partial pressure of arterial O_2_ (PaO_2_), (d) arterial oxygen saturation (SaO_2_), (e) arterial O_2_ content (CaO_2_), (f) partial pressure of arterial CO_2_ (PaCO_2_), (g) total arterial content of CO_2_ (CaCO_2_), (h) arterial bicarbonate ion concentration ([HCO_3_^−^]), (i) arterial hydrogen ion concentration ([H^+^]), and (j) cerebral perfusion pressure (CPP), depicted at baseline pre- and post-haemodilution. Asterisk (*) symbols indicate a change (P < 0.05) from pre to post haemodilution, compared using paired two-tailed t-tests. N = 11.

Isovolumic haemodilution reduced Hct (P < 0.001; Figure 1(b)) and [Hb] by 18 ± 2% (P < 0.001; Figure 1(a)). Since PaO_2_ (P = 0.32; Figure 1(c)) and SaO_2_ (P = 0.78; Figure 1(d)) were unchanged, the reduction in [Hb] resulted in an 18 ± 2% reduction in CaO_2_ (P < 0.001; Figure 1(e)). The PaCO_2_ was not different between pre- and post-haemodilution baselines (P = 0.21; Figure 1(f)), however, haemodilution caused reductions in arterial plasma CO_2_ content (see supplemental materials for calculations) (P < 0.001), total arterial content of CO_2_ (CaCO_2_) (P < 0.001; Figure 1(g)), and arterial [ ${\mathrm{HCO}}_{3}^{-}$ ] (P < 0.001; Figure 1(h)), while increasing arterial [H^+^] (P = 0.001; Figure 1(i)). There was no change in CPP (P = 0.84; Figure 1(k)), MAP (P = 0.465; Table 1), or jugular venous pressure (P = 0.810; Table 1) with haemodilution.

**Table 1.** Cerebrovascular parameters during CVR prior to and following haemodilution.

		Baseline	+3mmHg	+6mmHg	+9mmHg
MAP		*Haemodilution, P = 0.465;* ***CO_2_, P < 0.001*** *; Interaction, P = 0.860*			
(mmHg)	Pre	97.1 ± 4.8	99.4 ± 4.9	103.1 ± 5.6*†	107.6 ± 8.0*†‡
	Post	97.5 ± 5.3	99.8 ± 7.4	104.3 ± 7.9*†	108.6 ± 9.6*†‡
JVP		*Haemodilution, P = 0.810;* ***CO_2_, P < 0.001;*** *Interaction, P = 0.185*			
(mmHg)	Pre	6.4 ± 2.0	6.8 ± 1.9	7.2 ± 1.9*	7.8 ± 2.1*†‡
	Post	6.5 ± 0.9	6.7 ± 1.0	7.4 ± 1.5*	8.2 ± 1.9*†‡
ICA diameter		***Haemodilution, P = 0.042; CO_2_, P < 0.001*** *; Interaction, P = 0.479*			
(mm)	**Pre**	**5.26 ± 0.37**	5.35 ± 0.42*	5.47 ± 0.37*†	5.49 ± 0.38*†
	Post	5.16 ± 0.37	5.26 ± 0.36*	5.34 ± 0.34*†	5.44 ± 0.38*†
ICA velocity		***Haemodilution, P < 0.001; CO_2_, P < 0.001;*** *Interaction, P = 0.650*			
(cm/s)	Pre	48.1 ± 9.6	55.0 ± 12.7*	64.4 ± 12.8*†	70.7 ± 13.3*†‡
	**Post**	**59.2 ± 10.9**	68.1 ± 15.0*	78.5 ± 16.0*†	83.6 ± 16.4*†‡
ICA flow		***Haemodilution, P < 0.001; CO_2_, P < 0.001*** *; Interaction, P = 0.710*			
(mL/min)	Pre	314.4 ± 65.1	374.2 ± 98.2*	457.8 ± 96.5*†	507.1 ± 108.3*†‡
	**Post**	**376.5 ± 89.1**	448.0 ± 105.0*	531.6 ± 117.6*†	590.1 ± 144.2*†‡
ICA Shear rate		***Haemodilution, P < 0.001; CO_2_, P < 0.001;*** *Interaction, P = 0.602*			
(s^−1^)	Pre	368 ± 82	414 ± 101*	473 ± 109*†	516 ± 103*†‡
	**Post**	**460 ± 84**	520 ± 123*	589 ± 128*†	616 ± 124*†‡
ICA Shear stress		*Haemodilution, P = 0.089;* ***CO_2_, P < 0.001;*** *Interaction, P = 0.217*			
(dyne/cm^2^)	Pre	12.9 ± 3.2	14.5 ± 3.8*	16.6 ± 4.2*†	18.1 ± 3.9*†‡
	Post	12.4 ± 2.5	14.0 ± 3.4*	15.8 ± 3.6*†	16.5 ± 3.5*†‡
VA diameter		*Haemodilution, P = 0.506;* ***CO_2_, P < 0.001; Interaction, P = 0.002***			
(mm)	Pre	4.06 ± 0.31	4.08 ± 0.37	4.14 ± 0.33	4.20 ± 0.34*†
	Post	3.95 ± 0.23	3.99 ± 0.27	4.13 ± 0.29*†	4.26 ± 0.27*†‡
VA velocity		***Haemodilution, P < 0.001; CO_2_, P < 0.001;*** *Interaction, P = 0.640*			
(cm/s)	Pre	23.7 ± 7.6	26.8 ± 8.9	32.3 ± 10.8*†	36.9 ± 12.8*†‡
	**Post**	**28.5 ± 7.7**	**32.0 ± 8.2**	38.8 ± 11.8*†	43.7 ± 11.2*†‡
VA flow		***Haemodilution, P = 0.004; CO_2_, P < 0.001;*** *Interaction, P = 0.146*			
(mL/min)	Pre	93.4 ± 32.4	105.5 ± 34.2	131.8 ± 46.9*†	157.0 ± 64.2*†‡
	**Post**	**105.4 ± 29.9**	**120.7 ± 34.3**	157.9 ± 52.9*†	188.5 ± 54.3*†‡
VA Shear rate		***Haemodilution, P < 0.001; CO_2_, P < 0.001;*** *Interaction, P = 0.967*			
(s^−1^)	Pre	233.7 ± 74.9	264.4 ± 93.9	312.6 ± 106.1*†	349.7 ± 115.6*†‡
	**Post**	**289.3 ± 80.9**	**321.9 ± 83.8**	376 ± 114.5*†	411.4 ± 106.6*†‡
VA Shear stress		*Haemodilution, P = 0.220;* ***CO_2_, P < 0.001;*** *Interaction, P = 0.536*			
(dyne/cm^2^)	Pre	8.1 ± 2.4	9.2 ± 3.1	10.9 ± 3.4*†	12.2 ± 3.9*†‡
	Post	7.9 ± 2.5	8.8 ± 2.7	10.3 ± 3.3*†	11.3 ± 3.2*†‡
MCAv		***Haemodilution, P = 0.016; CO_2_, P < 0.001;*** *Interaction, P = 1.0*			
(cm/s)	Pre	70.9 ± 12.8	79.4 ± 16.1*	89.2 ± 19.1*†	99.4 ± 17.4*†‡
	**Post**	**77.3 ± 14.2**	85.8 ± 15.9*	95.8 ± 19.3*†	106.1 ± 21.1*†‡
PCAv		***Haemodilution, P = 0.008; CO_2_, P < 0.001;*** *Interaction, P = 0.194*			
(cm/s)	Pre	42.2 ± 7.4	47.0 ± 8.0*	52.1 ± 10.6*†	56.8 ± 12.6*†‡
	**Post**	**50.3 ± 14.4**	54.6 ± 15.5*	58.2 ± 14.2*†	60.6 ± 13.2*†‡
gCBF		***Haemodilution, P < 0.001; CO_2_, P < 0.001;*** *Interaction, P = 0.099*			
(mL/min)	Pre	833.1 ± 147.7	984.9 ± 233.6*	1187.7 ± 252.7*†	1333.2 ± 308.8*†‡
	**Post**	**987.1 ± 202.4**	1148.5 ± 241.5*	1391.8 ± 268.1*†	1585.8 ± 310.9*†‡

Comparisons conducted using linear mixed-model analyses with Bonferroni adjustments for post-hocs. Asterisk (*) symbols indicate a difference from baseline (P < 0.05) in both trials, obelisk (†) symbols indicate a difference from the +3 mm Hg stage (P < 0.05) in both trials, and double dagger (‡) symbols indicate a difference from the +6 mm Hg stage (P < 0.05) in both trials. Differences between pre and post haemodilution across all stages are identified by bolded type.

MAP: mean arterial pressure; JVP: jugular venous pressure; ICA: internal carotid artery; VA: vertebral artery; MCAv: middle cerebral artery blood velocity; PCAv: posterior cerebral artery blood velocity; gCBF: global cerebral blood flow.

#### Viscosity, shear rates and stresses

At all measured shear rates, whole blood viscosity was lower following haemodilution (45 s^−1^; 4.2 ± 0.3 vs. 3.1 ± 0.4 cP, P < 0.001: 90 s^−1^; 3.9 ± 0.3 vs. 2.9 ± 0.3 cP, P < 0.001: 225 s^−1^; 3.5 ± 0.2 vs. 2.8 ± 0.2 cP, P < 0.001). Basal ICA and VA shear rates were both higher post-haemodilution (368.2 ± 82.4 vs. 459.5 ± 84.0 s^−1^, P < 0.001; and 233.7 ± 74.9 vs. 289.3 ± 80.9 s^−1^, P = 0.004, respectively). The decreased viscosity and increased shear rates resulted in ICA and VA *shear stress* being unchanged from baseline (12.9 ± 3.2 vs. 12.4 ± 2.5 dyne/cm^2^, P = 0.26; and 8.1 ± 2.4 vs. 7.9 ± 2.5 dyne/cm^2^, P = 0.68, respectively).

#### Cerebral blood flow and metabolism parameters

**Figure 2.**
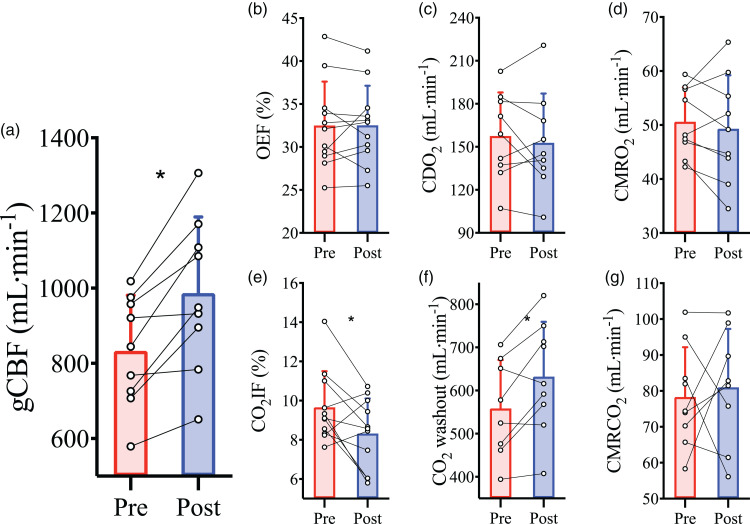
Cerebral blood flow and metabolism at baseline prior to and following haemodilution. Pre-haemodilution mean and standard deviation data presented in red, post-haemodilution mean and standard deviation data presented in blue, with individual data overlaid for both. (a) global cerebral blood flow (gCBF), (b) O_2_ extraction fraction (OEF), (c) cerebral delivery of O_2_ (CDO_2_), (d) cerebral metabolic rate of O_2_ consumption (CMRO_2_), (e) CO_2_ insertion fraction (CO_2_IF), (f) cerebral washout of CO_2_ content (CO_2_ washout) and (g) cerebral metabolic rate of CO_2_ production (CMRCO_2_), at baseline pre- and post-haemodilution. Asterisk (*) symbols indicate a change (P < 0.05) from pre to post haemodilution, compared using paired two-tailed t-tests. N = 9 for a, c, d, f, and g; while n = 11 for b and e.

Haemodilution caused an elevation in basal gCBF (833 ± 148 vs. 987 ± 202 ml/min; P = 0.002; Figure 2(a)). This increase in gCBF served to maintain CDO_2_ (P = 0.49; Figure 2(c)) in the face of the reduced CaO_2_ in order to preserve metabolism (cerebral metabolic rate of O_2_ (CMRO_2_); P = 0.49; Figure 2(d): cerebral metabolic rate of CO_2_ (CMRCO_2_); P = 0.57; Figure 2(g)) and oxygen extraction fraction (OEF) (p = 0.99; Figure 2(b)). The rise in gCBF and the maintenance of metabolism resulted in elevated CO_2_ washout (P = 0.01; Figure 2(f)), which was also reflected in the decrease of fraction of CO_2_ inserted into jugular blood (CO_2_IF; see supplemental materials for calculations) (P = 0.04; Figure 2(e)).

#### NO exchange

There was a positive cerebral a–v difference (25.9 ± 30.1 nM; P = 0.023; one-sided t-test) and net uptake (11.6 ± 14.0 nmol/min; P = 0.027; one-sided t-test) for plasma $NO_{2}^{-}$ prior to haemodilution but no a–v difference (−7.2 ± 34.1 nM; P = 0.52; one-sided t-test) or net uptake (−3.6 ± 17.9 nmol/min; P = 0.54; one-sided t-test) following haemodilution. However, when comparing pre- to post-haemodilution there was no difference in the a–v difference (P = 0.069; Supplemental Figure 2A) or net exchange (P = 0.098; Supplemental Figure 2B) of plasma $NO_{2}^{-}$ (paired t-tests) despite the mean change from uptake to release for each metric. Therefore, haemodilution appeared to eliminate the net cerebral consumption of plasma $\;NO_{2}^{-}$ observed at baseline.

#### Repeated measures correlations

We conducted repeated measures correlations assessing within-participant relationships between gCBF and parameters known to influence CBF, using values taken at baseline, mid-haemodilution checkpoint, and post-haemodilution baseline. There were intra-individual relationships between gCBF and: CaO_2_ (R^2^ = −0.44, P = 0.007), Hb (R^2^ = −0.45, P = 0.006), Hct (R^2^ = −0.44, P = 0.007), pH (R^2^ = −0.32, P = 0.029), and CaCO_2_ (R^2^ = −0.38, P = 0.015). As PaCO_2_ was clamped between stages and unchanged by haemodilution, there was no relationship with gCBF (P = 0.31) or CPP (P = 0.43).

### Cerebrovascular reactivity, pre and post haemodilution

#### Intervention parameters

By design, PaCO_2_ was elevated during the CVR test at each stage of hypercapnia (*CO_2_ stage*, P < 0.001) and each stage was matched between pre- and post-haemodilution (*trial*, P = 0.414; Supplemental Figure 3 A). The PaO_2_ was matched between pre- and post-haemodilution at each of stage of hypercapnia (*trial*, P = 0.342; Supplemental Figure 3B), while CaO_2_ was lower following haemodilution (*trial*, P < 0.001; Supplemental Figure 3 C). During CVR, neither MAP, JVP, nor CPP were different pre- versus post-haemodilution (*trial*, P = 0.465, P = 0.810, and P = 0.581, respectively), though all were elevated from baseline at the +6 and +9 mmHg PaCO_2_ stages (*CO_2_ stage,* all P < 0.001; Table 1).

#### Cerebral blood flow

**Figure 3.**
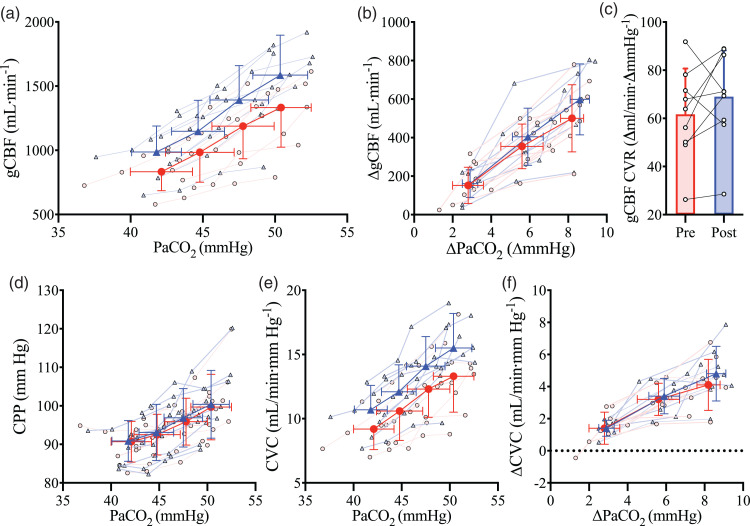
The relationship between global cerebral blood flow and arterial carbon dioxide. Mean and standard deviation for pre- (red circles/bar) and post- (blue triangles/bar) haemodilution, with individual data for both. (a) global cerebral blood flow (gCBF) at four stages of partial pressure of arterial CO_2_ (PaCO_2_), (b) gCBF changes from baseline with changes in PaCO_2_ from baseline, (c) cerebrovascular reactivity of gCBF (gCBF CVR) pre and post haemodilution, (d) cerebral perfusion pressure (CPP) at four stages of PaCO_2_, (e) cerebral vascular conductance (CVC) at four stages of PaCO_2_ and (f) CVC changes from baseline with changes in PaCO_2_ from baseline. N = 9.

Hypercapnia led to an increase in gCBF at each PaCO_2_ stage (*CO_2_ stage*, P < 0.001; Figure 3(a)). Post-haemodilution, gCBF was elevated at each stage compared to pre-haemodilution (*trial*, P < 0.001; Figure 3(a) and Table 1). To account for this is in the subsequent determination of CVR, we also compared gCBF change from baseline, which abolished the gCBF pre- versus post-haemodilution difference (*trial,* P = 0.198, Figure 3(b)). Shear stress did not differ between the pre- and post-haemodilution CVR trials (Table 1).

#### Cerebrovascular reactivity

While absolute gCBF was elevated throughout post-haemodilution compared to pre-haemodilution, the gCBF CVR response to PaCO_2_ was not different between trials (P = 0.24; Figure 3(c)), nor was gCBF CVR calculated using absolute delta gCBF (P = 0.14), or percent delta gCBF (P = 0.77). Similarly, CVR did not differ when gCBF was indexed against jugular venous partial pressure of CO_2_ (PjvCO_2_; P = 0.39), CaCO_2_ (P = 0.10), or jugular venous content of CO_2_ (CjvCO_2_) (P = 0.87), further demonstrating that CVR was not differentially affected by haemodilution at each stage of PaCO_2_. Finally, CVR of MCAv and PCAv were also unchanged (P = 0.59 and P = 0.25, respectively) with haemodilution. Table 2 presents all CVR slopes indexed against PaCO_2_.

**Table 2.** Cerebrovascular reactivity slopes in response to elevations in PaCO_2_.

	Absolute reactivity (X/mm Hg)			Absolute delta reactivity([ Δ X]/mm Hg)			Relative reactivity ([% Δ X]/mm Hg)		
	Pre	Post	P-value	Pre	Post	P-value	Pre	Post	P-value
ICA flow (mL/min)	23.9 ± 6.9	24.7 ± 7.9	0.76	23.9 ± 7.7	24.2 ± 10.4	0.92	7.7 ± 2.9	6.4 ± 2.4	0.22
VA flow (mL/min)	7.9 ± 4.2	9.3 ± 4.2	0.26	9.4 ± 7.5	11.6 ± 6.1	0.28	10.4 ± 4.6	11.4 ± 5.4	0.62
gCBF (mL/min)	61.7 ± 19.0	69.0 ± 19.2	0.23	65.7 ± 21.7	74.0 ± 23.6	0.14	7.8 ± 2.2	7.6 ± 2.8	0.77
MCAv (cm/s)	3.6 ± 0.8	3.2 ± 1.7	0.59	3.6 ± 0.6	3.3 ± 1.9	0.76	5.3 ± 0.9	4.8 ± 2.7	0.56
PCAv (cm/s)	1.7 ± 0.8	1.2 ± 0.8	0.25	1.8 ± 1.1	1.1 ± 1.0	0.25	4.1 ± 2.2	2.5 ± 1.7	0.16
gCBF CVC (mL/min/mmHg)	0.51 ± 0.17	0.56 ± 0.17	0.47	0.52 ± 0.21	0.58 ± 0.24	0.15	5.7 ± 2.5	5.5 ± 2.7	0.57

Comparisons conducted using paired two-tailed t-tests.

ICA: internal carotid artery; VA: vertebral artery; gCBF: global cerebral blood flow; MCAv: middle cerebral artery blood velocity; PCAv: posterior cerebral artery blood velocity; gCBF CVC: global cerebral blood flow cerebrovascular conductance.

#### Metabolism parameters

**Figure 4.**
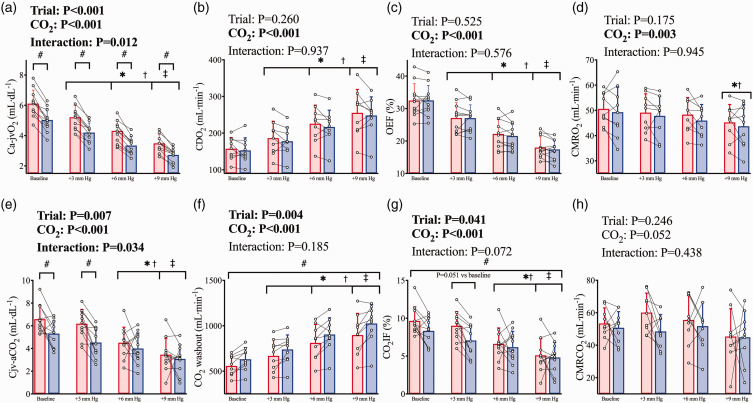
Cerebral metabolism during hypercapnia prior to and following haemodilution. Pre-haemodilution mean and standard deviation data presented in red, post-haemodilution mean and standard deviation data presented in blue, with individual data overlaid for both. (a) cerebral arteriovenous O_2_ content difference (Ca-jvO_2_), (b) cerebral O_2_ delivery (CDO_2_), (c) O_2_ extraction fraction (OEF), (d) cerebral metabolic rate of oxygen consumption (CMRO_2_), (e) cerebral veno-arterial CO_2_ content difference (Cjv-aCO_2_), (f) cerebral washout of CO_2_ content (CO_2_ washout), (g) CO_2_ insertion fraction (CO_2_IF), and (h) cerebral metabolic rate of CO_2_ production (CMRCO_2_). Comparisons conducted using linear mixed-model analyses with Bonferroni adjustments for post-hocs. Asterisk (*) symbols indicate a difference from baseline (P<0.05) in both trials, obelisk (†) symbols indicate a difference from the +3 mm Hg stage (P < 0.05) in both trials, and double dagger (‡) symbols indicate a difference from the +6 mm Hg stage (P < 0.05) in both trials. While hash symbols (#) indicate a difference (P < 0.05) between pre and post haemodilution in all CVR stages. Where an interaction effect is indicated, these symbols refer to changes between stages within trials, and differences between trials within stages. N = 11 for A, C, E, and G; while n = 9 for b, d, f, and h.

The Ca-jvO_2_ difference was lower post-haemodilution (*trial*, P < 0.001), as a result of the reduction in CaO_2_, and was narrowed at each step increase in PaCO_2_ (*CO_2_ stage,* P < 0.001) as a result of the increase in gCBF at each stage. There was a significant interaction effect (trial*CO_2_, P = 0.012) whereby during both pre- and post-haemodilution Ca-jvO_2_ was reduced (all P < 0.001) at each stage, and post-haemodilution Ca-jvO_2_ was lower than pre-haemodilution within each CVR stage (all P < 0.001; Figure 4(a)). Despite the reduced CaO_2_, the overall elevation in gCBF post-haemodilution served to maintain CDO_2_ comparably to pre-haemodilution throughout the CVR test (*trial,* P = 0.260; Figure 4(b)). As such, OEF was also unchanged between trials (*trial*, P = 0.525) and lower at each stage of CVR compared to baseline (*CO_2_ stage*, P < 0.001; Figure 4(c)). CMRO_2_ was not different between trials (*trial* P = 0.175) and was lower (*CO_2_ stage*, P < 0.001; Figure 4(d)) at the +9 mm Hg PaCO_2_ stage compared to baseline (P = 0.002) and +3 mm Hg (P = 0.034) stages. When determined as the change from baseline, a difference between trials remained for Ca-jvO_2_ (*trial*, P = 0.041).

**Figure 5.**
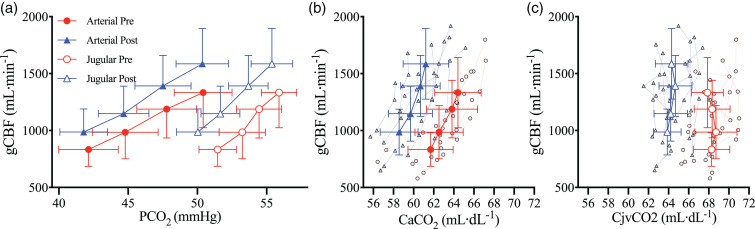
Cerebral CO_2_ tensions and contents during hypercapnia prior to and following haemodilution. Solid red circles and blue triangles represent arterial blood pre and post haemodilution, respectively, while empty red circles and blue triangles represent jugular venous blood pre and post haemodilution, respectively. (a) global cerebral blood flow (gCBF) against the partial pressures of CO_2_ in arterial (PaCO_2_) and jugular venous (PjvCO_2_) bloods, (b) gCBF against the arterial content of CO_2_ (CaCO_2_), and (c) gCBF against the jugular venous content of CO_2_ (CjvCO_2_). All data are mean and standard deviations, with individual data overlaid for b, c, and d. N = 9.

During CVR, the Cjv-aCO_2_ difference was lower post-haemodilution (trial*CO_2_, P = 0.034) at baseline (P = 0.004) and +3 mmHg PaCO_2_ (P < 0.001) stages but not +6 or +9 mmHg stages (Figure 4(e)). The overall elevation in gCBF post-haemodilution elevated CO_2_ washout across all stages (*trial,* P = 0.004), and the expected CVR response elevated gCBF in tandem with the elevated PaCO_2_ at each stage, thereby increasing absolute CO_2_ washout at each stage (*CO_2_ stage* P < 0.001; Figure 4(f)). Correspondingly, CO_2_IF was lower post-haemodilution (*trial*, P = 0.041) and reduced at each step increase in PaCO_2_ (*CO_2_ stage*, P < 0.001; Figure 4(g)). CMRCO_2_ was maintained between trials (*trial* P = 0.246) and was nominally unchanged with hypercapnia (*CO*_2_ *stage,* P = 0.052; Figure 4(h)). When determined as the change from baseline, the interaction effect remained for Cjv-aCO_2_ (trial*CO_2_, P = 0.026), but the effect of trial was abolished for CO_2_IF (*trial*, P = 0.385) and CO_2_ washout (*trial*, P = 0.366). Finally, while PaCO_2_ and PjvCO_2_ were both elevated at each stage of CVR (Figure 5(a)), the rise in CaCO_2_ (Figure 5(b)) was not matched with a corresponding rise in total CO_2_ content of the cerebral effluent (CjvCO_2_). Indeed, although CjvCO_2_ was reduced following haemodilution (*trial*, P < 0.001), it was unchanged across PaCO_2_ stages in either trial (*CO_2_ stage*, P = 0.190; Figure 5(c)).

## Discussion

The purpose of this study was to investigate the influence of acute isovolumic haemodilution on CBF and cerebrovascular reactivity to CO_2_ in healthy humans. Cerebrovascular regulation was preserved following haemodilution, indicated by an unaltered CVR. Furthermore, CBF increased following haemodilution to an extent adequate to preserve CDO_2_ and thereby maintain the requirements of CMRO_2_, which also resulted in increased CO_2_ washout. This preservation of cerebrovascular function coincided with an abolishment of cerebral net uptake of ${\mathrm{NO}}_{2}^{-}$ . Overall, our findings indicate that in the setting of acute experimental anaemia in the absence of pathology, cerebrovascular metabolism and regulation by CO_2_ are preserved.

Along with the principal regulators of CBF [i.e., blood pressure, metabolism, blood gases^1,3,6,49,50^] a number of other factors have been implicated in CBF control. For example, both animal and human studies have reported effects of changes in blood viscosity, Hct, [Hb], and NO bioavailability on CBF.^26,32,33,51–56^ Based on these studies, we hypothesized that haemodilution would steepen the CVR response. However, our findings demonstrate CVR is unchanged with haemodilution (Figure 3), which is in agreement with previous rodent studies,^25^ and with Tu & Liu^16^ who found that following acute isovolumic haemodilution in healthy humans, CBF was elevated while cerebrovascular reactivity to acetazolamide was reduced in the cortex but not in white matter, the putamen, or thalamus (differences in stimulus and mechanisms acknowledged). Below, we place the related findings in the context of the contributory roles of viscosity, Hct, [Hb], and NO and highlight the implications of elevations in CBF on central pH balance.

### Regulation of CBF in the setting of acute anaemia

Following the removal and replacement of whole blood with albumin (a non-oxygen-carrying protein), Hct was reduced by 20%, which caused an approximate 20% reduction in CaO_2_ (Figure 1). Concomitant with this mild anaemia, gCBF was elevated by approximately 18% (Figure 2). While it has been well established that CBF is inversely related to CaO_2_^10,^^51^ indicating the increase in basal CBF following anaemia was likely driven by hypoxic vasodilation, other factors that may concurrently influence CBF cannot be disregarded. Indeed, in addition to the reductions in [Hb] and CaO_2_ (Figure 1(a) and (e), respectively), haemodilution was also associated with an increase in arterial [H^+^] and a reduction in arterial [ ${\mathrm{HCO}}_{3}^{-}$ ] (Figure 1(i) and (h), respectively). Much work has been presupposed on the notion that arterial [H^+^] is a predominant driver of CBF, although other work contends that in fact PaCO_2_ plays the primary role, diffusing across the blood brain barrier to alter perivascular PCO_2_ and thus [H^+^].^57–61^ Evidence also suggests that [ ${\mathrm{HCO}}_{3}^{-}$ ] may independently influence CBF.^57,62^ Our results can neither confirm nor contest this as our study design does not allow for differentiation of the effects of changes in [H^+^] from the effects of changes in CaO_2_. However, recent data from our laboratory does indicate that acute changes in arterial [H^+^] independent of PaCO_2_ are not associated with concurrent alterations in CVR.^57^ Nevertheless, while the reduction in CaO_2_ was likely the primary factor elevating basal CBF following haemodilution, other factors cannot be wholly discounted, as outlined next.

As the reduction in CaO_2_ coincided with reduced Hct, there is further potential for alterations in blood viscosity *per se –* consequent to the changes in Hct - to alter CBF regulation. In rodents, extreme elevations in whole blood viscosity substantially affect CBF during haemodilution and hypercapnia^52^ and exhaust available vasodilator reserve, thereby abolishing hypercapnic CVR.^55^ Yet, *in vivo* in humans, viscosity appears to have a lesser role in CBF regulation compared to the effects of changes in CaO_2_.^51,63^ For example, to isolate the changes in whole blood viscosity from CaO_2_, Brown and Marshall^56,63^ investigated the influence of a reduction in blood viscosity secondary to plasmapheresis of paraproteinemic patients on CBF. The authors observed no change in CBF following a large reduction in plasma viscosity, and thus whole blood viscosity with which there was no accompanying change in CaO_2_;^56,63^ this is consistent with animal studies showing the greater degree of contribution of CaO_2_ to changes in CBF during haemodilution.^18^ Further, haemodilution-induced anaemia has been shown to increase CBF to a much lesser extent than carbon monoxide inhalation,^12^ despite the decreased blood viscosity associated with haemodilution that is absent with carbon monoxide inhalation [acknowledging the potential for the confound of direct vasodilator influences of carbon monoxide].^64^

The majority of studies assessing the effects of hematocrit on CVR have been conducted in animals^25,30,53,65,66^ or in humans in the clinical setting of cardiovascular pathology.^54^ Relative to human investigations, while Ševerdija et al.^54^ found no difference in hypercapnic CVR between high and low hematocrit groups ( ≥ 28% vs < 28%), varied methods make reconciling the findings of these studies with our own difficult. For instance, Ševerdija et al.^54^ report CVR as the percent change in cerebral tissue (or rather Hb) oxygen saturation assessed via near-infrared spectroscopy per change in PaCO_2_ during hypercapnic and hypocapnic steps induced via mechanical ventilation. These CVR tests were conducted during anaesthesia with Propofol and sufentanil, for coronary bypass surgery, in elderly patients. However, it has been demonstrated that there is poor agreement between near-infrared spectroscopy and measures of volumetric CBF during hypercapnia.^44^ Additionally, the comparisons in that study were between groups, rather than within-individuals, and could be confounded by the between individual variability typical of physiologic measures especially in elderly patients with cardiovascular pathology, hindering the relevance to the current study. Ultimately, however, the findings in the study by Ševerdija et al.^54^ and our findings are consistent. While analogous considerations apply, previous work in baboons demonstrated that CVR varied inversely with hematocrit,^30^ supporting our findings that show acute reductions in Hct are associated with elevated gCBF.

It is known that Hb is a powerful scavenger of NO,^32,67–69^ thus reductions in Hb could hypothetically affect CBF through reduced scavenging^32^ and improved NO bioavailability. Equivalent findings relative to the peripheral circulation have recently been demonstrated, i.e., NO-dependent shear-mediated dilation of the brachial artery was augmented following a haemodilution induced reduction in Hb.^26,27^ While the notion of a reduction in Hb scavenging of NO was a notable aspect of our hypothesis building, the contribution of NO to CVR remains a contentious topic. Reviews on the summarized evidence of CVR regulatory mechanisms^6,70–72^ and combined recent investigations^33,73–76^ suggest that while NO plays a fundamental role in basal CBF regulation, it has a merely permissive rather than obligatory role during hypercapnia in humans (though evidence to the contrary also exists^77,78^). In other words, while hypercapnia stimulates cerebrovascular NO production^33^ and downstream secondary messenger activity,^79^ inhibition of this NO production has no influence on the CVR response.^33^ As such, any reductions in Hb NO scavenging may plausibly have had some effect on CBF during baseline, but minimal or no impact on responses during CVR. Indeed, given arterial-to-venous differences of ${\mathrm{NO}}_{2}^{-}$ reflect endothelial NO production and are related to endothelium dependent vasodilation,^33,80^ the abolishment of net cerebral ${\mathrm{NO}}_{2}^{-}$ uptake from pre- to post-haemodilution may represent a reduction in Hb scavenging of NO and a mechanism of the increase in CBF post haemodilution.

Haemodilution decreased viscosity, and in conjunction with a concomitant increase in shear rate at each stage, shear stress was unaltered pre-to-post-haemodilution at each stage of hypercapnia. While supra-physiologically increased viscosity exhausts available cerebrovascular vasodilator reserve and thereby abolishes CVR in rats,^55^ the modest reduction in arterial viscosity *per se* during haemodilution would be unlikely to *steepen* CVR responses. Conversely, it may be expected that the anaemia-induced elevation of CBF might blunt CVR through encroaching on cerebrovascular reserve.^20^ However, CVR was not blunted, and – at least according to our volumetric measures - the haemodilution-induced elevation in gCBF did not exhaust cerebrovascular reserve in the large arteries (e.g. ICA and VA diameters were reduced and unchanged, respectively, following haemodilution; Table 1). While anaemia demanded CBF elevation in order to maintain CDO_2_ (Figure 2) presumably via vasodilation at the level of the pial arteries and microcirculation, this was achieved without vasodilation of the large arteries (relative to pre-haemodilution), but rather through faster velocities (Table 1) while the decrease in viscosity meant that cerebral vascular shear stress was maintained at roughly pre-haemodilution magnitude (Table 1).

### Teleological perspective

The elevation in CBF with haemodilution was associated with an increase in CO_2_ washout (Figure 2), which is relevant to maintaining central pH.^4,5^ From a teleological perspective, in the setting of acute anaemia, elevated basal gCBF suffices to not only maintain CDO_2_, but also likely maintains central pH. The finding of unchanged CjvCO_2_ across all stages of CVR during both trials (Figure 5) further supports this conception. That total CO_2_ in the cerebral effluent blood is unchanged with increased PaCO_2_ is evidence of the efficacy of CVR mechanisms; indeed, it belies the function of CVR to maintain cerebral tissue acid-base balance. Whether or not the impaired CVR observed in anaemic pathologies is related to a disturbance in CO_2_ washout or central pH remains unknown. In the absence of a concomitant increase in gCBF, the reduction in the CO_2_ carrying capacity of the blood with isovolumic haemodilution would have resulted in build-up of CO_2_ causing cerebral tissue acidosis. Further, whether the contemporaneous increase in CO_2_ washout was merely coincidental or in fact a contributing influence in the increase in basal gCBF is also unknown.

### Experimental considerations

We only studied male participants. Inclusion of both sexes is vital to a full understanding of the roles of NO, Hb, Hct, and viscosity in CBF regulation and for physiological research generally.^81^ Thus the results of the current study cannot be extrapolated to females and future research is needed to address this limitation.^81^ Abundant opportunities are available for future research regarding this matter to assess possible sex-related differences, the influence of androgens and estrogens, across the female menstrual cycle, pre versus post menopause, and pre versus post puberty. Furthermore, the sample size was relatively small compared to some of the larger clinical trials assessing the effect of anemia on CBF, and as such may be underpowered for the comparisons of some variables.

We acknowledge that as a variable for the assessment of cerebrovascular function, total content of CO_2_ likely only has significance in settings where some aspect of blood CO_2_ transport (i.e., Hb, [H^+^], [HCO_3_^−^], etc.) is altered – e.g., anaemia or other pathologies, high altitude, exercise, etc. During single CVR tests, calculation of total CO_2_ content will provide little benefit over PaCO_2_, however in this study, it provided insight into the underlying factors allowing for the maintenance of CVR. Finally, the degree of anemia achieved in this investigation was relatively mild compared to that found in some severe pathologies, as such our findings may not hold true for more moderate or severe anemias. Nonetheless, mild anemia in clinical populations has been associated with impaired CVR compared to healthy controls, with hemoglobin concentrations comparable to the current study (e.g. 10.3 g/dL in chronic renal failure patients; our study, 11.3 g/dL post-hemodilution).^21^ Thus, in those instances of mild anaemia where CO_2_ reactivity is impaired, our findings suggest it is likely impaired not because of anaemia *per se*, but other pathologic factors associated with the disease (e.g. perhaps a hemolytic component and cell free Hb are more important in determining impairments in CVR in pathologically chronic anaemia).

## Conclusion

Acute experimental anaemia increased gCBF to maintain CDO_2_ and preserve CMRO_2_; this led to – or was in conjunction with – the maintenance of hypercapnic CVR. Therefore, we conclude that in the setting of acute experimental anaemia, absent pathology, cerebral metabolism and the CBF response to reduced CaO_2_ are robust enough to also allow for normal vascular regulation by CO_2_. This indicates that reductions of CVR seen in pathology are more likely the result of the direct vascular effects of disease states rather than due to the effects of anaemia *per se*.

## Abbreviations

CaCO_2_ – arterial content of arterial CO_2_

CaO_2_ – arterial content of O_2_

CBF – cerebral blood flow

CDO_2_ – cerebral delivery of O_2_

CjvCO_2_ – jugular venous content of CO_2_

CMRCO_2_ – cerebral metabolic rate of CO_2_

CMRO_2_ – cerebral metabolic rate of O_2_

CO – cardiac output

CO_2_ – carbon dioxide

CPP – cerebral perfusion pressure

CVR – cerebrovascular reactivity to CO_2_

DEF – dynamic end-tidal forcing

gCBF – global cerebral blood flow

H^+^ – hydrogen ions

Hb – hemoglobin

HCO_3_^−^ – bicarbonateions

Hct – hematocrit

HR – heart rate

ICA – internal carotid artery

JVP – jugular venous pressure

MAP – mean arterial pressure

MCAv –middle cerebral artery blood velocity

${\mathrm{NO}}_{2}^{-}$ – nitrite

NO – nitric oxide

O_2_ – oxygen

OEF – oxygen extraction fraction

PaCO_2_ – arterial partial pressure of CO_2_

PaO_2_ – arterial partial pressure of O_2_

PCAv – posterior cerebral artery blood velocity

P_ET_CO_2_ – end-tidal partial pressure of CO_2_

P_ET_O_2_ – end-tidal partial pressure of O_2_

PjvCO_2_ – jugular venous partial pressure of CO_2_

PjvO_2_ – jugular venous partial pressure of O_2_

RSNO – *S*-nitrosothiols

SaO_2_ – arterial O_2_ saturation

TCD – transcranial doppler ultrasound

VA – vertebral artery

## Supplemental Material

sj-pdf-1-jcb-10.1177_0271678X221119442 - Supplemental material for Cerebral O_2_ and CO_2_ transport in isovolumic haemodilution: Compensation of cerebral delivery of O_2_ and maintenance of cerebrovascular reactivity to CO_2_ Click here for additional data file. Supplemental material, sj-pdf-1-jcb-10.1177_0271678X221119442 for Cerebral O_2_ and CO_2_ transport in isovolumic haemodilution: Compensation of cerebral delivery of O_2_ and maintenance of cerebrovascular reactivity to CO_2_ by Jay MJR Carr, Philip N Ainslie, David B MacLeod, Joshua C Tremblay, Daniela Nowak-Flück, Connor A Howe, Mike Stembridge, Alexander Patrician, Geoff B Coombs, Benjamin S Stacey, Damian M Bailey, Daniel J Green and Ryan L Hoiland in Journal of Cerebral Blood Flow & Metabolism

## Data Availability

The data that support the findings of this study are available from the corresponding author upon reasonable request. Supplementary material for this paper can be found at the journal website: http://journals.sagepub.com/home/jcb.
