# Supplementary material for: Cerebral O2 and CO2 transport in isovolumic haemodilution: Compensation of cerebral delivery of O2 and maintenance of cerebrovascular reactivity to CO2
Source: J Cereb Blood Flow Metab. 2022 Sep 21;43(1):99–114. doi: 10.1177/0271678X221119442 (PMC9875354; doi:10.1177/0271678X221119442)
Supplement: sj-pdf-1-jcb-10.1177_0271678X221119442 - Supplemental material for Cerebral O2 and CO2 transport in isovolumic haemodilution: Compensation of cerebral delivery of O2 and maintenance of cerebrovascular reactivity to CO2 [file sj-pdf-1-jcb-10.1177_0271678X221119442.pdf]

Calculations

Volumetric cerebral blood flow was calculated as follows:

Equation 1: ICA or VA flow (mL/min) = $\left(\frac{1}{2}\cdot\text{Peak Envelope Velocity}\right) \times \left(\pi\left(\frac{1}{2}\cdot\text{diameter}\right)^2\right) \times 60$

Global CBF (gCBF) was calculated as twice the sum of our unilateral ICA and VA measurements, acknowledging potential limitations [1]:

Equation 2: gCBF (mL/min) = 2 × (ICA flow + VA flow).

CVR was determined by the slope of the relationships between gCBF and $PaCO_2$ (repeated with MCAv and PCAv). Shear stress was calculated as the product of shear rate and whole blood viscosity measured at 225 s^{-1}:

Equation 3: shear stress ($dyne/cm^2$) = shear rate (s^{-1}) · (whole blood viscosity (cP) / 100).

To account for cerebral perfusion pressure (CPP = MAP – jugular venous pressure) in our analyses of the CBF responses, cerebrovascular conductance (CVC) was subsequently calculated:

Equation 4: $\text{CVC (mL/min} \cdot \text{mm Hg}^{-1}) = \frac{\text{gCBF (mL/min)}}{\text{CPP (mm Hg)}}$

CVR of CVC was determined by the slope of the relationship between CVC and $PaCO_2$. Oxygen extraction fraction (OEF) - defined as the fraction of O_2 extracted from the arterial blood - is expressed as a percentage, where an increase can reflect either elevated O_2 consumption, diminished O_2 delivery, or both, and conversely a decrease of OEF can reflect either reduced O_2 consumption, increased O_2 delivery, or both:

Equation 5: $\text{OEF (\%)} = \frac{\text{CaO}_2\text{ (mL/dL)} - \text{CvO}_2\text{ (mL/dL)}}{\text{CaO}_2\text{ (mL/dL)}} \cdot 100$

Cerebral delivery of oxygen (CDO_2):

Equation 6: $\text{CDO}_2\text{ (mL/min)} = \frac{\text{gCBF (mL/min)}}{1000} \cdot [\text{CaO}_2\text{(mL/dL)} \cdot 10]$

Cerebral metabolic rate of oxygen consumption ($CMRO_2$) was calculated:

Equation 7: $$CMRO_2\ (mL/min) = \frac{gCBF\ (mL/min)}{1000} \cdot \{[CaO_2\ (mL/dL) - CvO_2\ (mL/dL)] \cdot 10\}$$

CO_2 in blood is transported in three main modes; freely dissolved (i.e. that which is measured as PCO_2), and bound as both bicarbonate (HCO_3^-), and as carbamate to Hb ($HbCO_2$), and it is the latter two by which the majority of CO_2 is carried [2–6]. As such, isovolumic haemodilution would certainly cause not only reduced CaO_2, but also the reduction of total CO_2 carrying capacity due to reduced $[HCO_3^-]$ and both erythrocyte-bound and cell-free Hb. Furthermore, at sites such as the capillaries where CO_2 diffuses freely across the vascular wall the appropriate measure is surely as a partial pressure, however, since dehydration and rehydration of CO_2 is catalytically accelerated by carbonic anhydrase to ~2 milliseconds [4], at sites such as large vessels where blood is conveyed away from tissues and diffusional areas, the partial pressure of CO_2 represents only a fraction of the total CO_2 being transported. As such, we assessed cerebral CO_2 parameters by calculating total arterial and cerebral venous CO_2 content (CCO_2) per Douglas *et al.* (1988) for comprehensive blood gas interpretation of the changes incurred by haemodilution. First, we calculated the plasma content of CO_2 ($PCCO_2$):

Equation 8: $$PaCCO_2 \text{ or } PvCCO_2 = 2.226 \cdot s \cdot PCO_2 \cdot (1 + 10^{pH - pK`})$$

Where *s* is the solubility coefficient of CO_2 and pK` is the apparent pK [both calculated per Kelman (1967), with the assumption that core temperature was 37.5°C and unchanged with haemodilution]. $PCCO_2$ was calculated for both arterial ($PCaCO_2$) and jugular venous ($PCjvCO_2$) blood using arterial pH, $PCO_2$, and pK` for $PCaCO_2$ and jugular venous pH, PCO_2, and pK` for $PCjvCO_2$. Then, CCO_2:

Equation 9: $$CaCO_2 \text{ or } CvCO_2 = PCCO_2 \cdot \frac{(1 - 0.0289 \cdot [Hb])}{(3.352 - 0.456 \text{ x } SO_2) \cdot (8.142 - pH)}$$

Where SO_2 is O_2 saturation. CCO_2 was calculated for both arterial ($CaCO_2$) and jugular venous ($CjvCO_2$) blood, using arterial Hb, SO_2, and pH for $CaCO_2$ and jugular venous Hb, SO_2, and pH for $CjvCO_2$. Then, manipulation of the Fick equation with substitution of CaO_2 and $CjvO_2$ with $CjvCO_2$ and $CaCO_2$, respectively, allowed for the calculation of the CO_2 insertion fraction (CO_2IF), defined here as the CO_2 deposited into the jugular venous blood from the cerebral tissues, expressed as a percentage (analogous and inverse to oxygen extraction fraction). An increase in CO_2IF can reflect either elevated CO_2 production,

diminished CO_2 washout, or both; and, conversely, a decrease of CO_2IF can reflect either reduced CO_2 production, increased CO_2 washout, or both:

Equation 10: $$CO_2IF\ (\%) = \frac{CjvCO_2\ (mL/dL) - CaCO_2\ (mL/dL)}{CjvCO_2\ (mL/dL)} \cdot 100$$

We also calculated cerebral CO_2 washout [in the supine position, jugular venous flow represents ~95% of total cerebral outflow [9,10]; as such, we used arterial gCBF (mL/min) in place of global cerebral venous blood flow]:

Equation 11: $$CO_2\ washout\ (mL/min) = \frac{gCBF\ (mL/min)}{1000} \cdot [CjvCO_2\ (mL/dL) \cdot 10]$$

As well as the cerebral metabolic rate of CO_2 production ($CMRCO_2$):

Equation 12: $$CMRCO_2\ (mL/min) = \frac{gCBF\ (mL/min)}{1000} \cdot \{[CjvCO_2\ (mL/dL) - CaCO_2\ (mL/dL)] \cdot 10\}$$

As another indicator of cerebral CO_2 status, we took $CjvCO_2$ to be an index of cerebral tissue CO_2. Although the majority of work published to date has utilized PCO_2 values [whether jugular venous, the cerebral a-v difference, or the arithmetic mean+1 [11]] as indicators of cerebral tissue or cerebrospinal fluid CO_2, given that cerebral tissue CO_2 status will change not only with $PaCO_2$, but also with CBF, intracellular $[H^+]$, $[HCO_3$-], and tissue metabolism, and also for the reasons given above for calculation of CO_2 content, we believe that CO_2 content in the cerebral effluent is a suitable marker of cerebral tissue CO_2 in this particular setting.

Given the previous work demonstrating changes in [Hb] may influence NO bioavailability / signal transduction [12–14], we also calculated trans-cerebral exchange of plasma NO_2^-:

Equation 13: $$Plasma\ NO_2^-\ exchange\ (nmol/min) = \frac{gCBF\ (mL/min)}{1000} \cdot (1-\frac{Hct}{100}) \cdot [arterial\ NO_2^-\ (nM) - jugular\ venous\ NO_2\ (nM)]$$

Where positive values indicate cerebral net *uptake*, and negative values indicate net *release*.

Supplemental figures:

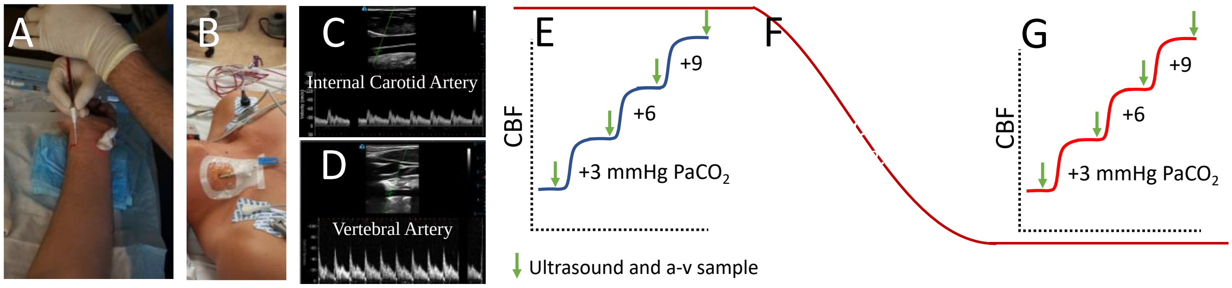

Supplemental Figure 1. Protocol schematic

A, B, C, and D represent aspects of instrumentation and measurement (A, placement of radial arterial catheter, B placement of internal jugular venous bulb catheter, C and D are screen captures of internal carotid artery and vertebral artery respectively). E, F, and G represent the protocol; i.e., cerebrovascular reactivity to CO_2 (3 stages of elevated $PaCO_2$) followed by haemodilution via blood removal and replacement with Albumin, targeting a ~20% reduction of blood volume, followed by post-haemodilution CVR. Aspects of the diagram have been reproduced with permission from [15].

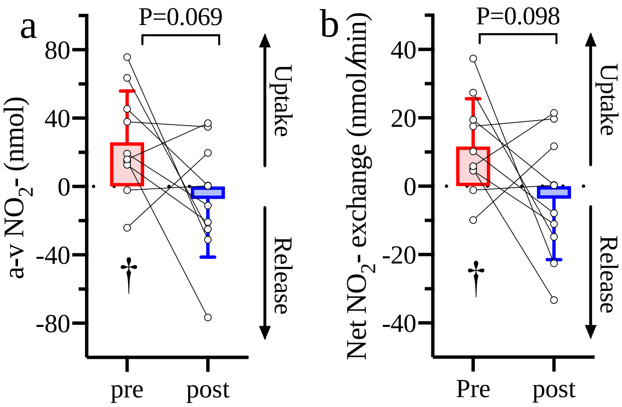

Supplemental Figure 2. Trans-cerebral plasma nitrite exchange prior to and following haemodilution.

Panel A depicts the arterial-to-jugular venous (a-v) difference for plasma nitrite (NO_2^-), while Panel B depicts the cerebral net exchange for plasma NO_2^-. An obelisk (†) symbol reflects a significant a-v difference or net exchange of plasma NO_2^- (one-sided t-test). Pre- to post-haemodilution comparisons were conducted with paired t-tests. N=10.

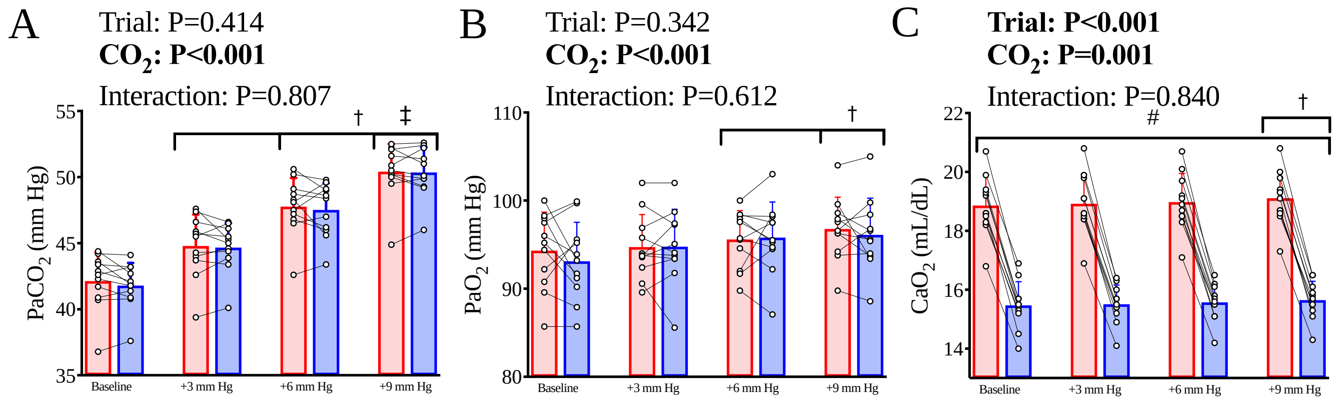

Supplemental Figure 3. Blood gases during hypercapnia prior to and following haemodilution.

Pre-haemodilution mean and standard deviation data presented in red, post-haemodilution mean and standard deviation data presented in blue, with individual data overlaid for both. A, partial pressure of arterial CO_2 ($PaCO_2$), B, partial pressure of arterial O_2 (PaO_2), C, arterial O_2 content (CaO_2). Comparisons conducted using linear mixed-model analyses with Bonferroni adjustments for post-hocs. Asterisk (*) symbols indicate a difference from baseline (P<0.05) in both trials, obelisk (†) symbols indicate a difference from the +3 mm Hg stage (P<0.05) in both trials, and double dagger (‡) symbols indicate a difference from the +6 mm Hg stage (P<0.05) in both trials. While hash symbols (#) indicate a difference (P<0.05) between pre and post haemodilution in all CVR stages. N=11

1. Friend AT, Rogan M, Rossetti GMK, et al. Bilateral regional extracranial blood flow regulation to hypoxia and unilateral duplex ultrasound measurement error. *Exp Physiol* 2021; 106: 1535–1548.
2. Margaria R. The contribution of hemoglobin to acid-base equilibrium of the blood in health and disease. *Clinical chemistry* 1957; 3: 306.
3. Margaria R. On the state of CO2 in blood and haemoglobin solutions, with an appendix on some osmotic properties of glycine in solution. *J Physiol* 1931; 73: 311.
4. Geers C, Gros G. Carbon dioxide transport and carbonic anhydrase in blood and muscle. *Physiol Rev* 2000; 80: 681–715.
5. Roughton FJW. RECENT WORK ON CARBON DIOXIDE THE BLOOD TRANSPORT BY THE BLOOD. *Physiol Rev* 1935; 15: 241-296.
6. van Slyke DD. The carbon dioxide carriers of the blood. *Physiol Rev* 1921; 1: 141–176.
7. Douglas AR, Jones NL, Reed JW. Calculation of whole blood CO2 content. *J Appl Physiol* 1988; 65: 473–477.
8. Kelman GR. Digital computer procedure for the conversion of PCO2 into blood CO2 content . *Respiration physiology* 1967; 3: 111–115.
9. Gisolf J, van Lieshout JJ, van Heusden K, et al. Human cerebral venous outflow pathway

depends on posture and central venous pressure. *J Physiol* 2004; 560: 317–327.
10. Manuel Valdueza J, von Münster T, Hoffman O, et al. Postural dependency of the cerebral venous outflow. *The Lancet (British edition)* 2000; 355: 200–201.
11. Fencl V. Acid-Base Balance in Cerebral Fluids. In: *Handbook of Physiology, The Respiratory System II, Control of Breathing*. 1986, pp. 115–140.
12. Tremblay JC, Hoiland RL, Howe CA, et al. Global REACH 2018: High Blood Viscosity and Hemoglobin Concentration Contribute to Reduced Flow-Mediated Dilation in High-Altitude Excessive Erythrocytosis. *Hypertens (Dallas, Tex 1979)* 2019; 73: 1327–1335.
13. Hoiland RL, Tremblay JC, Stacey BS, et al. Acute reductions in haematocrit increase flow-mediated dilatation independent of resting nitric oxide bioavailability in humans. *J Physiol* 2020; 598: 4225–4236.
14. Azarov I, Huang KT, Basu S, et al. Nitric Oxide Scavenging by Red Blood Cells as a Function of Hematocrit and Oxygenation. *J Biol Chem* 2005; 280: 39024–39032.
15. Hoiland RL, Caldwell HG, Carr JMJ. R, et al. Nitric oxide contributes to cerebrovascular shear-mediated dilatation but not steady-state cerebrovascular reactivity to carbon dioxide. *J Physiol* 2021; 0: 1–19.
